# Dialysis Water Supply Faucet as Reservoir for Carbapenemase-Producing *Pseudomonas aeruginosa*

**DOI:** 10.3201/eid2810.220731

**Published:** 2022-10

**Authors:** Christopher Prestel, Heather Moulton-Meissner, Paige Gable, Richard A. Stanton, Janet Glowicz, Lauren Franco, Mary McConnell, Tiffany Torres, Dijo John, Gillian Blackwell, Renae Yates, Chavela Brown, Kristina Reyes, Gillian A. McAllister, Jasen Kunz, Erin E. Conners, Katharine M. Benedict, Amy Kirby, Mia Mattioli, Kerui Xu, Nicole Gualandi, Stephanie Booth, Shannon Novosad, Matthew Arduino, Alison Laufer Halpin, Katherine Wells, Maroya Spalding Walters

**Affiliations:** Centers for Disease Control and Prevention, Atlanta, Georgia, USA (C. Prestel, H. Moulton-Meissner, P. Gable, R.A. Stanton, J. Glowicz, L. Franco, G.A. McAllister, J. Kunz, E.E. Conners, K.M. Benedict, A. Kirby, M. Mattioli, K. Xu, N. Gualandi, S. Booth, S. Novosad, M. Arduino, A. Laufer Halpin, M.S. Walters);; City of Lubbock Health Department, Lubbock, Texas, USA (M. McConnell, T. Torres, D. John, K. Wells);; Texas Department of State Health Services, Austin, Texas (G. Blackwell);; University Medical Centers, Lubbock (R. Yates, C. Brown, K. Reyes)

**Keywords:** Pseudomonas aeruginosa, healthcare-associated infections, nosocomial infections, antimicrobial resistance, multidrug-resistant organisms, carbapenemase-producing bacteria, premise plumbing, bacteria, Texas, United States

## Abstract

During June 2017–November 2019, a total 36 patients with carbapenem-resistant *Pseudomonas aeruginosa* harboring Verona-integron–encoded metallo-β-lactamase were identified in a city in western Texas, USA. A faucet contaminated with the organism, identified through environmental sampling, in a specialty care room was the likely source for infection in a subset of patients.

Verona-integron–encoded metallo-β-lactamase–producing carbapenem-resistant *Pseudomonas aeruginosa* (VIM-CRPA) and other carbapenemase-producing organisms (CPOs) are emerging public health threats. CPOs cause infections that are often extensively drug-resistant and associated with substantial rates of illness and death. By colonizing faucet aerators and wastewater plumbing systems, CPOs can spread rapidly within healthcare facilities, including to patients (*1*–*9*). VIM is a carbapenemase, a type of enzyme that inactivates carbapenems and other β-lactam antimicrobial drugs that are frequently encoded on mobile genetic elements, which in turn can lead to horizontal spread. 

VIM-CRPA is uncommon in the United States; <150 isolates are reported to CDC annually (*10*). During June 2017–November 2019, in a city of 250,000 residents in western Texas, USA (city A), 36 patients with VIM-CRPA were identified. Most were hospitalized for >1 night at an acute-care hospital (hospital A) in the 6 months before VIM-CRPA was isolated, but patients did not have overlapping hospital stays or common procedures. We assessed water sources and plumbing in hospital A to identify potential VIM-CRPA reservoirs. 

Beginning in June 2017, the Texas Department of State Health Services asked clinical laboratories to voluntarily submit clinical *P. aeruginosa* isolates resistant to imipenem, meropenem, or doripenem to the Texas Department of State Health Services Laboratory for mechanism testing through the Antibiotic Resistance Laboratory Network (https://www.cdc.gov/drugresistance/ar-lab-networks/domestic.html). The 2 clinical laboratories that served hospitals in city A began submitting all CRPA for mechanism testing in June 2017 (hospital A) and April 2018 (hospital B). During July 2017–January 2019, a total of 36 patients with VIM-CRPA isolated from clinical cultures were identified from city laboratories; 21 (58%) had been admitted to hospital A for >1 night in the 6 months before culture collection. ­­

We reviewed medical records from hospital A of patients with VIM-CRPA. Median patient age was 57 (range 9–84) years; 57% were male. VIM-CRPA was isolated from wounds in 9 (43%) patients, respiratory sources in 7 (33%), and urine in 5 (24%). Most patients primarily received care on either medical/surgical units (n = 13, 62%) or intensive care units (ICU 1 or ICU 2; n = 6, 29%). Among persons who had been hospitalized at hospital A in the previous 6 months, VIM-CRPA–positive specimens collected by hospital day 2 were classified as healthcare-associated, community-onset (n = 11, 52%); those collected on or after the third hospitalization day were considered hospital-onset (n = 10, 48%). No patients overlapped on the same unit at the same time, but 3 were placed in the same room in ICU 1, room A, over a 2-year period. A single point-prevalence screening of patients in ICU 1 in October 2018 did not identify additional *P. aeruginosa*–colonized patients. On the basis of common exposure to room A, we considered the potential for an environmental reservoir.

## The Study

We conducted an environmental investigation focused on water supplies and other sites conducive to biofilm formation. We collected 85 samples from plumbing fixtures and environmental surfaces in patient care areas as well as from water intake and storage areas, and evaluated for the presence of VIM-CRPA (Appendix). 

**Figure 1:**
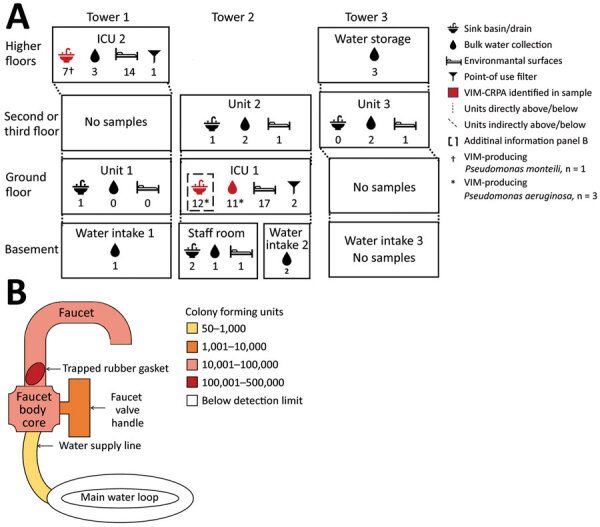
Environmental sampling scheme at hospital A from a study of CRPA in acute-care hospital specialty care unit, Texas, USA. A) Collection location in hospital and number of each sample type (icons with numbers underneath) collected (N = 85). We selected units and rooms for environmental sampling on the basis of chart review, focusing on where patients who developed clinical infections were located; patient rooms were those where patients with VIM-CRPA had been previously located. Three patients developed clinical infections while in ICU 1 and 3 while in ICU 2. Thirteen other patients from several medical or surgical units also developed clinical infections. Samples from which we recovered >1 VIM-producing isolate are indicated in red. We identified VIM-CRPA from 3 sites related to a single sink in room A of ICU 1: the sink drain, the interior surface of the dialysis faucet, and bulk water from a dialysis faucet used as the water source for the reverse osmosis unit of portable dialysis machines. We identified VIM-producing *Pseudomonas monteilii* (†) in a single sink basin sample of 1 room in ICU 2. B) Schematic view and heatmap of colony forming units identified by culture at selected internal surface locations within the faucet and water supply used for portable dialysis in ICU 1, room A. CRPA, carbapenem-resistant *Pseudomonas aeruginosa*; ICU, intensive care unit; VIM, Verona-integron-encoded metallo-β-lactamase.

We identified VIM-CRPA in 3 sites in room A related to a sink: the drain, bulk water (1 L of tap water), and the interior surface of a faucet serving as the water source for the reverse osmosis unit of portable dialysis machines (Figure 1, panel A). Of note, this faucet did not have an aerator, which has been implicated as a source of contamination in prior outbreaks. We did not recover VIM-CRPA from the interior of a second faucet intended for hand hygiene, the bulk water or point-of-use filter from that faucet, surface samples of the sink basin, or nearby areas. 

The dialysis faucet in room A was installed in October 2017. After we identified VIM-CRPA from associated samples, it was disassembled, revealing a rubber gasket trapped in the gooseneck. We collected swab samples from the interior of the disassembled faucet, valve, and core; we then instilled water into the fixture, agitated it using a sonic device, and filtered it onto culture medium with 4 rinses from the faucet, connection supply line, core, and gasket. We cultured VIM-CRPA from the gasket, faucet, and water supply line (Figure 1, panel B). Three patients with VIM-CRPA infections received care in room A over a 6-month period; 1 had an infection identified on hospital day 46 and was discharged 1 month before the dialysis faucet was installed. None of the infected patients received dialysis.

We considered that portable dialysis machines attachable to the contaminated faucet could spread VIM-CRPA to other dialysis hook-ups and sink drains where effluent reverse osmosis water was discharged. We cultured bulk water and swab samples from the sink in the biomedical room where dialysis machines were cleaned, the dialysis machine connector, and the tubing from the reverse osmosis unit of the dialysis machine connected to the contaminated faucet to test for CPOs, but identified no VIM-CRPA. After removing the dialysis faucet and adopting measures intended to mitigate spread of organisms from premise plumbing (*11*), we identified no additional VIM-CRPA clinical cultures in patients admitted to the specialty unit. 

**Figure 2:**
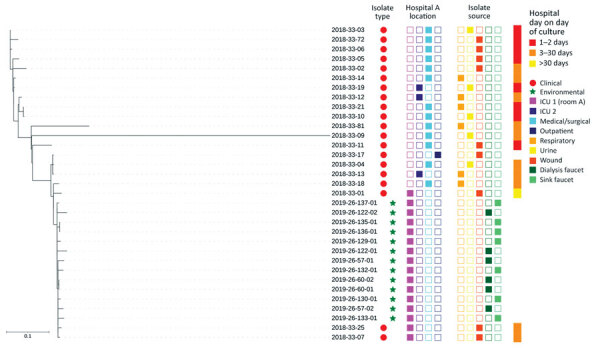
Whole-genome sequencing dendrogram of VIM-CRPA clinical (N = 20) and environmental (N = 13) isolates from hospital A, Texas, USA. Location of culture collection, isolate source, and patient hospital day when clinical culture was obtained are shown. All isolates were sequence type 308 and harbored a VIM-2 allele. No hospital day is provided for isolate 2018-33-17 because it was collected during an emergency department encounter; patient had had an overnight hospitalization in hospital A 2 weeks earlier. ICU, intensive care unit.

We performed whole-genome sequencing on 33 VIM-CRPA clinical isolates from 20 patients at multiple locations in hospital A and 13 environmental isolates from room A (Figure 2; Appendix); sequences are available at the National Center for Biotechnology Information (BioProject ID PRJNA288601). All 33 isolates were sequence type 308 and harbored VIM-2; clinical isolates varied by 3–255 (median 54, mean 60.3) high-quality single-nucleotide variants (hqSNVs) (*12*–*14*). Environmental isolates from the faucet and clinical isolates from 3 patients admitted to room A varied from 0–24 (median 10, mean 11.8) hqSNVs. The hqSNVs were derived from a conserved core of 6.5 Mb, which covered on average 91% of the assembled genome for the 33 isolates in the analysis.

The mechanism by which the faucet became contaminated is unknown. We considered it might have been through the water supply, considering a recent report of carbapenemase-producing organisms, although not VIM-producing, in US municipal water systems (*15*); however, none of the 24 water samples collected from other hospital locations grew any CPOs in cultures. Given the stay in room A of a patient with VIM-CRPA before the faucet was installed, we hypothesize that the sink drain became contaminated first, followed by retrograde contamination from the sink drain to the faucet, either during installation or through patient care activities. Although the misplaced rubber gasket provided a nidus for contamination, whether that was necessary for persistent faucet contamination is unclear. 

We could not ascertain relative contributions of the faucet interior and upper part of the sink drain toward patients acquiring VIM-CRPA. Although none of the patients underwent dialysis, the faucet was not labeled for dialysis use and may have been used for hand hygiene and other purposes. Infection prevention efforts at hospital A focused on improving sink hygiene, including removing patient care supplies from sink splash zones and regularly cleaning splash zones to prevent future transmission to patients from wastewater plumbing. During the 18-month period after the sink hygiene interventions began in October 2018, another 2 cases were identified, but in units unrelated to the specialty care unit. 

## Conclusions

We identified VIM-CRPA from a dialysis faucet, in water from that faucet, and from the associated sink drain in an ICU room where VIM-CRPA infections of the same strain developed in 3 patients. Although healthcare facility wastewater plumbing and faucet aerators are well-documented reservoirs of CPOs, our findings highlight the importance of considering other plumbing sources as well. 

Appendix Additional information about investigation of carbapenemase-producing *Pseudomonas aeruginosa* in acute care hospital specialty care unit, Texas, USA.

## References

[1] Decraene V, Phan HTT, George R, Wyllie DH, Akinremi O, Aiken Z, et al.; TRACE Investigators’ Group. A large, refractory nosocomial outbreak of *Klebsiella pneumoni*ae carbapenemase-producing *Escherichia coli* demonstrates carbapenemase gene outbreaks involving sink sites require novel approaches to infection control. Antimicrob Agents Chemother. 2018;62:e01689–18. 10.1128/AAC.01689-18 30249685 PMC6256751

[2] Salm F, Deja M, Gastmeier P, Kola A, Hansen S, Behnke M, et al. Prolonged outbreak of clonal MDR *Pseudomonas aeruginosa* on an intensive care unit: contaminated sinks and contamination of ultra-filtrate bags as possible route of transmission? Antimicrob Resist Infect Control. 2016;5:53. 10.1186/s13756-016-0157-9 27980730 PMC5139016

[3] Kanamori H, Weber DJ, Rutala WA. Healthcare outbreaks associated with a water reservoir and infection prevention strategies. Clin Infect Dis. 2016;62:1423–35. 10.1093/cid/ciw122 26936670

[4] Hopman J, Meijer C, Kenters N, Coolen JPM, Ghamati MR, Mehtar S, et al. Risk assessment after a severe hospital-acquired infection associated with carbapenemase-producing *Pseudomonas aeruginosa.* JAMA Netw Open. 2019;2:e187665. 10.1001/jamanetworkopen.2018.7665 30768189 PMC6484879

[5] Hajar Z, Mana TSC, Cadnum JL, Donskey CJ. Dispersal of gram-negative bacilli from contaminated sink drains to cover gowns and hands during hand washing. Infect Control Hosp Epidemiol. 2019;40:460–2. 10.1017/ice.2019.25 30767838

[6] Knoester M, de Boer MG, Maarleveld JJ, Claas EC, Bernards AT, de Jonge E, et al. An integrated approach to control a prolonged outbreak of multidrug-resistant *Pseudomonas aeruginosa* in an intensive care unit. Clin Microbiol Infect. 2014;20:O207–15. 10.1111/1469-0691.12372 24707852

[7] Aranega-Bou P, George RP, Verlander NQ, Paton S, Bennett A, Moore G, et al.; TRACE Investigators’ Group. Carbapenem-resistant *Enterobacteriaceae* dispersal from sinks is linked to drain position and drainage rates in a laboratory model system. J Hosp Infect. 2019;102:63–9. 10.1016/j.jhin.2018.12.007 PMC6504032 30571992

[8] Kotay S, Chai W, Guilford W, Barry K, Mathers AJ. Spread from the sink to the patient: *in situ* study using green fluorescent protein (GFP)–expressing *Escherichia coli* to model bacterial dispersion from hand-washing sink-trap reservoirs. Appl Environ Microbiol. 2017;83:e03327. 10.1128/AEM.03327-16 28235877 PMC5377511

[9] Chia PY, Sengupta S, Kukreja A, S L Ponnampalavanar S, Ng OT, Marimuthu K. The role of hospital environment in transmissions of multidrug-resistant gram-negative organisms. Antimicrob Resist Infect Control. 2020;9:29 . 10.1186/s13756-020-0685-1 32046775 PMC7014667

[10] Centers for Disease Control and Prevention. Antibiotic resistance [cited 2021 Sep 19]. https://arpsp.cdc.gov/profile/antibiotic-resistance

[11] Centers for Disease Control and Prevention. Healthcare-associated infections: reduce risk from water: from plumbing to patients [cited 2022 Feb 8]. https://www.cdc.gov/hai/prevent/environment/water.html

[12] Stanton RA, Vlachos N, Laufer Halpin A. GAMMA: a tool for the rapid identification, classification, and annotation of translated gene matches from sequencing data. Bioinformatics. 2022;38:546–8. 10.1093/bioinformatics/btab607 34415321

[13] Petkau A, Mabon P, Sieffert C, Knox NC, Cabral J, Iskander M, et al. SNVPhyl: a single nucleotide variant phylogenomics pipeline for microbial genomic epidemiology. Microb Genom. 2017;3:e000116. 10.1099/mgen.0.000116 29026651 PMC5628696

[14] Letunic I, Bork P. Interactive Tree Of Life (iTOL) v5: an online tool for phylogenetic tree display and annotation. Nucleic Acids Res. 2021;49(W1):W293–6. 10.1093/nar/gkab301 33885785 PMC8265157

[15] Tanner WD, VanDerslice JA, Goel RK, Leecaster MK, Fisher MA, Olstadt J, et al. Multi-state study of *Enterobacteriaceae* harboring extended-spectrum beta-lactamase and carbapenemase genes in U.S. drinking water. Sci Rep. 2019;9:3938. 10.1038/s41598-019-40420-0 PMC6408426 30850706

