# Supplementary material for: Dialysis Water Supply Faucet as Reservoir for Carbapenemase-Producing Pseudomonas aeruginosa
Source: Emerg Infect Dis. 2022 Oct;28(10):2069–73. doi: 10.3201/eid2810.220731 (PMC9514332; doi:10.3201/eid2810.220731)
Supplement: Appendix — Additional information about investigation of carbapenemase-producing Pseudomonas aeruginosa in acute care hospital specialty care unit, Texas, USA. [file 22-0731-Techapp-s1.pdf]

Article DOI: https://doi.org/10.3201/eid2810.220371

Contaminated Dialysis Water Supply Faucet as Reservoir for Carbapenemase-Producing *Pseudomonas aeruginosa*

Appendix

Laboratory methods

Environmental samples at Hospital A included one-liter samples of first-catch and post-flush water (bulk water), swab samples of faucet interiors, drain covers, sink drains, and sink basins, sponge stick samples from sink basins and from countertops within the one-meter splash zone of sinks and point-of-use filters (Pall-Aquasafe; https://www.pall.com) were collected from sampled faucets when present. Surface environmental samples were collected using Amies Transport Media Swabs or Sponge-Stick with neutralizing buffer. Bulk 1L water samples were collected after five-minute flush from a spigot. All samples were sent to CDC for further testing.

Environmental surface samples were placed directly into 10 mL of asparagine broth (made in-house), vortexed for 10 seconds, and incubated for 18–24 hours at 35°C. After incubation, 0.1 mL of turbid broth enrichment was plated onto CHROMagar *Pseudomonas* (made in-house) and MacConkey II agar (MAC; Becton, Dickinson and Co.; https://www.bd.com) and streaked for isolation. Bulk water samples underwent serial dilutions in Butterfield Buffer (BB, made in-house) and were plated onto Reasoner's 2 Agar (R2A, made in-house) and Tryptic Soy Agar (TSA, Becton-Dickinson). The remaining volume was filtered through a 0.22-micron pore size filter in 200 mL increments and then placed onto CHROMagar *Pseudomonas* media (https://www.chromagar.com). CHROMagar *Pseudomonas* cultures were incubated at 30°C and MAC cultures were incubated at 35°C. All plated cultures were screened for suspect colonies at 18–24 hours and incubated further for up to 72 hours for presence/absence of suspect colonies.

All bacterial isolates from environmental samples were identified by matrix-assisted desorption ionization time-of-flight mass spectrometry (MALDI-TOF MS) (MALDI Biotyper,

Bruker Daltonics; https://www.bruker.com) using the Bruker and MicrobeNet (https://microbnet.cdc.gov) databases. DNA extraction for the detection of carbapenemase genes was performed directly from broth enrichment using the Maxwell 16 and MDx Instruments (http://mdxinstruments.com). Detection of carbapenemase genes was performed using Applied Biosystems' Fast 7500 Real-Time Polymerase Chain Reaction (RT-PCR) System (Applied Biosystems; https://www.thermofisher.com) for bla_{VIM} from *Pseudomonas aeruginosa* isolates. DNA for whole genome sequencing was extracted from pure cultures using Maxwell MDx Instrument and genomic libraries were prepared using the PerkinElmer Zephyr G3 NGS Workstation (https://www.perkinelmer.com) and NuGEN Ovation Ultralow System V2 assay kit. Libraries were sequenced using an Illumina MiSeq System (https://www.illumina.com). Raw reads were processed using the QuAISAR-H pipeline (https://github.com/DHQP/QuAISAR_singularity/) and AR genes were called using GAMMA (https://github.com/rastanton/GAMMA). The phylogeny of the sequences was analyzed using SNVPhyl (https://github.com/phac-nml/snvphyl-galaxy) and the resultant tree was annotated using iTol (https://itol.embl.de). Isolate 2018–33–01 was used as the reference isolate for read mapping as it was the centroid genome of the set by Mash distances. Given the close genetic relationship between the VIM-CRPA isolates, detailed analysis to describe the genetic environment of the bla_{VIM-2} was not performed.
